# Symmetrical distributions of aminoacyl-tRNA synthetases during the evolution of the genetic code

**DOI:** 10.1007/s12064-023-00394-0

**Published:** 2023-07-05

**Authors:** Marco V. José, Juan R. Bobadilla, Gabriel S. Zamudio, Sávio Torres de Farías

**Affiliations:** 1 Theoretical Biology Group, Instituto de Investigaciones Biomédicas, Universidad Nacional Autónoma de México, CP 04510 Mexico City, Mexico; 2 Laboratório de Genética Evolutiva Paulo Leminsk, Departamento de Biologia Molecular, Universidade Federal da Paraíba, João Pessoa, Paraíba Brazil

**Keywords:** Amino-acyl-tRNA synthetases, Symmetry groups, Evolution genetic code, Hypercubes, Polytopes, RNY code, Extended RNA codes 1 and 2

## Abstract

In this work, we formulate the following question: How the distribution of aminoacyl-tRNA synthetases (aaRSs) went from an ancestral bidirectional gene (mirror symmetry) to the symmetrical distribution of aaRSs in a six-dimensional hypercube of the Standard Genetic Code (SGC)? We assume a primeval RNY code, two Extended Genetic RNA codes type 1 and 2, and the SGC. We outline the types of symmetries of the distribution of aaRSs in each code. The symmetry groups of aaRSs in each code are described, until the symmetries of the SGC display a mirror symmetry. Considering both Extended RNA codes the 20 aaRSs were already present before the Last Universal Ancestor. These findings reveal intricacies in the diversification of aaRSs accompanied by the evolution of the genetic code.

## Introduction

The understanding of the genetic code, its origin and evolution, remains as a fundamental problem in biology. Several informational molecules participate in the implementation of the genetic code, such as DNA, DNA polymerase, RNA polymerase, mRNA, tRNA, rRNA, amino acids, and aminoacyl-tRNA synthetases (aaRSs). There are specific interactions between these molecules, which involve transcription that results in mRNA and translation that produce proteins. There are several mathematical models aimed to model the genetic code and most of them focus on the mapping of 61 codons or triplets to 20 canonical amino acids. The genetic code includes three triplets that are stop signals. To our knowledge, theoretical mathematical works on the distribution of aaRSs in the genetic code are nonexistent. For example, there are mathematical models on the symmetries (Hornos and Hornos 1993), the optimality and evolution (Novozhilov et al. 2007; Zamudio and José 2018; Wnętrzak et al. 2018), the degeneracy (González et al. 2019), and the uniqueness (Zamudio and José 2017) of the genetic code.

Aminoacyl-tRNA synthetases (aaRSs) are essential enzymes that catalyze the esterification of a tRNA to its cognate L-amino acid (i.e., the amino acid corresponding to the anticodon of the tRNA according to the genetic code) (Ibba and SÖll 2000; Pang et al. 2014). AaRSs acylate the 3′-terminal CCA sequence with the cognate amino acid to produce aminoacyl-tRNA (aa-tRNAs) (Dale & Uhlenbeck 2005). Aa-tRNAs are carried to the ribosome as building blocks for protein synthesis by the elongation factors EF-Tu in bacteria, and EF1A in archaea and eukaryotes (Dale and Uhlenbeck 2005; Steitz 2008; Fahlman et al. 2004). AaRSs originated very early in evolution, and it is accepted that an almost complete set was already present in the last universal common ancestor (LUCA) (Nagel & Doolittle 1995; Woese et al. 2000; O’Donoghue and Luthey-Schulten 2003; Fournier et al. 2011). AaRSs are ancient enzymes whose amino acid specificity was generally established at the time of LUCA (Ribas de Pouplana et al. 2020). AaRSs are a unique family of enzymes, as they are the only proteins that can decode the rules of the genetic code, all while being translated following those same rules. Essentially, an aaRS exhibits double specificity, one for the amino acid and one for the cognate tRNA (operational code), i.e., the origin of the genetic code. These two specificities must have coevolved. In the case of GlyRS, it was shown that the anticodon stem loop contributed to the encoding system and just with the emergence of the mRNA it was co-opted for codification (Farias et al. 2019). In this model of the origin of the genetic code, the operational code evolved in conjunction with the anticodon code (Farias et al. 2019). There are 20 aaRSs, one for each of the 20 standard amino acids. AaRSs can be divided into two mutually exclusive classes, I and II, based on their structural, functional, and evolutionary relatedness (Ibba and SÖll 2000; Cusack et al. 1990; Eriani et al. 1990; Burbaum and Schimmel 1991; Ribas de Pouplana and Schimmel 2001a, 2001b; Schimmel et al. 1993). Each aaRS falls into either Class I or Class II, except for lysyl-tRNA synthetase (LysRS), which has a representative in both classes. Within each class, there exist subclasses a, b, and c based on relative sequence and structural similarity. Albeit the two classes do not seem to share common ancestors, it is believed that the ancient aaRSs constituted just the canonical core aminoacylation site and during evolution aaRSs acquired various domains including editing domains (Ribas de Pouplana and Schimmel 2001a, 2001b; Schimmel et al. 1993).

How mRNA translation evolved from a simple beginning to its complex and accurate contemporary state is unknown. The emergence of the genetic code from the operational RNA code could occur when the second domain of synthetases was added with the anticodon-containing domain of tRNAs (Schimmel et al. 1993).

The specificity of interactions between aaRS and tRNA during the transfer of amino acid and charging tRNA is mainly determined by two domains in tRNA, the minihelix at the acceptor stem and the anticodon loop (Woese et al. 2000; Schimmel et al. 1993; Ribas de Pouplana and Schimmel 2001a, 2001b).

The Rodin–Ohno (RO) model divides the table of the genetic code into two classes of aminoacyl-tRNA synthetases (Classes I and II) with recognition from the minor or major groove sides of the tRNA acceptor stem (Rodin and Ohno 1995, 2006, 2008; Rodin et al. 2009; Carter et al. 2014). According to the table of the genetic code, the RO model is almost symmetric. On the other hand, the standard genetic code (SGC) can be algebraically derived from the primeval RNY code (R stands for purines, Y for pyrimidines and N any of them) (José et al. 2007). The RO-model can also be derived from a primeval RNY code by means of group actions, namely symmetries represented by automorphisms (José et al. 2017). It turns out that the RO model is symmetric in a six-dimensional (6D) hypercube (José et al. 2017). Conversely, using the same automorphisms, the RO-model can lead to the SGC. Class aaRS I (Class aaRS II) can be converted into Class aaRS II (Class aaRS I) by means of isometric algebraic functions (José et al. 2017). The four polar requirement categories display a symmetrical arrangement in a 6D hypercube. Altogether, these results can be achieved thanks to the use of 4, 5, and 6 dimensions. Notably, the 6D algebraic model is compatible with both the SGC (based upon the primeval RNY code) and the RO-model (José et al. 2017).

AaRS are grouped into Classes I and II based on primary and tertiary structure and enzyme properties suggesting two independent phylogenetic lineages. Similarly, tRNA molecules can also form two respective classes, based on the class membership of their corresponding aaRS (Eriani et al. 1990). Besides the crucial role of aaRSs in protein synthesis, there is a plethora of other cellular functions (Ibba and SÖll 2000; Pang et al. 2014). Extensive structural and biochemical studies have shown that aaRS enzymes can be grouped in two different classes (I and II) based on sequence motifs, active site topology, tRNA binding and aminoacylation site (Ribas de Pouplana 2020; Cusack et al. 1990; Eriani et al. 1990; Burbaum and Schimmel 1991; Ribas de Pouplana and Schimmel 2001a, 2001b). Based on these findings, it is commonly assumed that the aaRSs are descendants of two ancestral enzymes. The two distinct classes exist in all three domains of life: Bacteria, Archaea and Eukarya (Eriani et al. 1990; Burbaum and Schimmel 1991; Ribas de Pouplana and Schimmel 2001a, 2001b; Schimmel, et al. 1993). Moreover, synthetases within each class can be further subdivided into subclasses of enzymes that tend to recognize chemically related amino acids (Eriani et al. 1990; Burbaum and Schimmel 1991; Ribas de Pouplana and Schimmel 2001a, 2001b).

Because aminoacylation of tRNAs establishes the current genetic code, it stands to reason that there was a close co-evolution of tRNAs and synthetases that started from a simpler, primordial mechanism presumably maintained by ribozymes. Experimental results (Caporaso et al. 2005; Hohn et al. 2006) and phylogenetic analyses (Rodin and Rodin 2006, 2008) support the view of takeover from ribozymes.

The fact that enzymes belonging to the two synthetase classes are grossly mirror images of each other (e.g., they approach opposite sides on tRNAs) motivated a phylogenetic analysis that indicated that these proteins were originally coded for by opposite strands of the same gene (Rodin and Rodin. 2006) in the later stages of the RNA world. This scenario was strengthened experimentally (Carter et al. 2014). Herein, we consider the hypothesis of a primeval RNA genetic code which consists of the 16 codons of type RNY. Using simple algebraic operations, we have shown how the RNA code could have evolved toward the current SGC via two different intermediate evolutionary stages called Extended RNA codes type 1 and 2 (José et al. 2011). By rotations or translations of the subset RNY, we arrive at the SGC via the former (type 1) or via the latter (type 2), respectively (José et al. 2011). Biologically, the Extended RNA code type 1 consists of all codons of the type RNY plus codons obtained by considering the RNA code but in the second (NYR type) and third (YRN type) reading frames (Table 1). The Extended RNA code type 2 comprises all codons of the type RNY plus codons that arise from transversions of the RNA code in the first (YNY type) and third (RNR) nucleotide bases. The SGC is obtained via the complementary set of codons RRR and YYY in the case of the Extended RNA code type 1, and/or the complementary set of codons YRR and YYR of the Extended RNA code type 2 (Table 1).

**Table 1:**
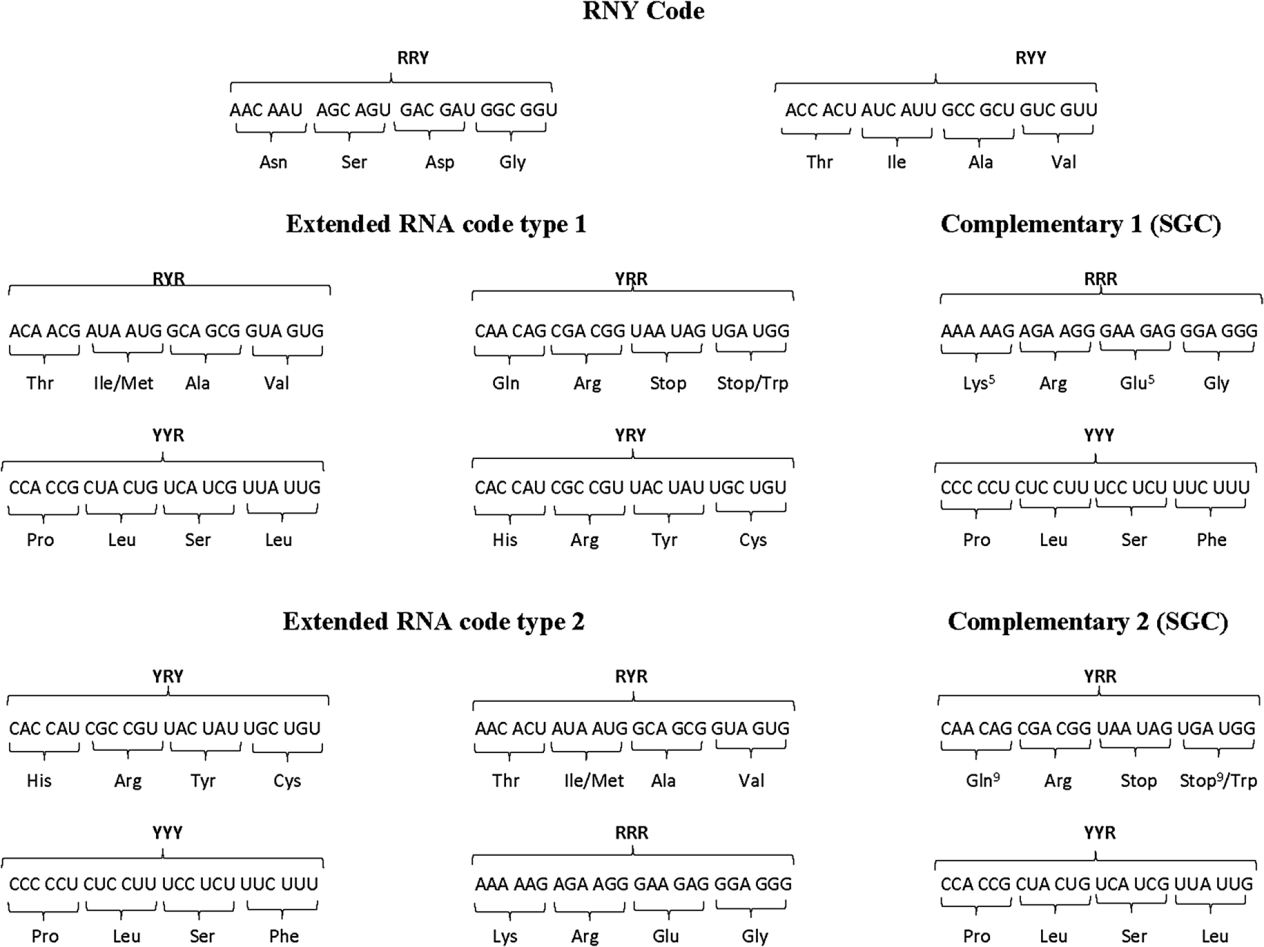
The standard genetic code

The Extended RNA code type 1 is obtained by transversions in the first and/or the third nucleotides of codons. The Extended RNA code type 2 is derived by frameshift reading mistranslations of codons. The SGC is achieved in each case, via the addition of their respective complementary codes.

In previous works, Genetic Hotels of codons and Hotels of amino acids (three-dimensional models) were built and whole genomes of bacteria and archaea were used to test hypotheses about the evolution of the SGC (José et al. 2011, 2009).

In this work, we pose the question: How the distribution of aaRSs went from the Rodin-Ohno hypothesis that two enzyme superfamilies descended from one ancestral gene (mirror symmetry) to the symmetrical distribution of aaRSs in the 6D representation of the SGC? We assume a primeval RNY code, two Extended Genetic RNA codes type 1 and 2 (José et al. 2017), and the SGC, and determine the types of symmetries of the distribution of aaRSs in each code. The evolutionary implications of our results are discussed.

The article is organized as follows: First, we show that for each of the sets RNY, YNY, RNR, and YNR there is an associated RNA code which can be represented as a 4-dimensional hypercube as derived by concepts of combinatorial geometry. Interestingly, the four 4-dimensional hypercubes can be inserted as pairwise disjoint 4-dimensional affine subspaces in the 6-dimensional hypercube (Coxeter 1973) of the SGC.

### Biological background

A little more than 20 aaRSs are found in modern organisms. They are classified into two groups, Class I and Class II, each having three subclasses (a–c) based on similarity in sequences and structures (Nagel and Doolittle 1995; Woese et al. 2000). The classification is as follows: Class Ia (MetRS, ValRS, LeuRS, IleRS, CysRS, and ArgRS); Class Ib (GluRS, GlnRS and LysRS-class I); Class Ic (TyrRS and TrpRS); class IIa (SerRS, ThrRS, AlaRS, GlyRS-α2, ProRS, and HisRS); Class IIb (AspRS, AsnRS, and LysRS-class II); and Class IIc (PheRS, GlyRS-α2β2, SepRS, and PylRS). In general, aaRS consists of a catalytic domain, an anticodon-binding domain, and often an editing domain. Each class harbors class-specific characteristic motifs and structural topology in its catalytic domains (Eriani et al. 1990).

According to the RO model (Rodin and Ohno 1995; Rodin and Rodin 2006, 2008; Rodin et al. 2009; Carter et al. 2014), the table of the genetic code can be divided into the sub-codes NAN, NGN, NUN, NCN. We have also shown that there exists an automorphism *F* of the cube defined also piecewise, which transforms that division into the sub-codes RNR, YNR, RNY, YNY, respectively, which is precisely our algebraic model (José et al. 2007, 2017, 2011).

The SGC has been modeled in a 6D hypercube using group theory (José and Zamudio 2020; Sethuraman 1997). The vertices of the hypercube represent the 64 possible nucleotide triplets and the edges join triplets that differ by one nucleotide under different arrangements of the nucleotides in a square (José and Zamudio 2020). Each of the three possible arrangements of the nucleotides in a square produces different orderings of the codon triplets in the hypercube (José and Zamudio 2020); a fourth arrangement of the nucleotides is given by the square with its two diagonals, representing a scenario where all possible nucleotide changes are within reach in one-step mutation. The four possible arrangements of the four nucleotides (A, C, G, U) as the vertices of a square are: {A, C, U, G}, {A, G, U, C}, {A, C, G, U}, and {A, C. G, U}.

### Mathematical background

Algebraic groups measure symmetry. The order of the automorphism group of an object is a crude measure of the amount of symmetry of the object; the structure of the automorphism group, and of its action on the object, gives us much more detailed information. A symmetry group is a group of symmetry-preserving operations, i.e., rotations, reflections, and inversions (Sethuraman 1997; Artin 1991; Weisstein 2022). For the Extended RNA code type 2 and the SGC, isometric functions that transform the Class aaRS I into the Class aaRS II and vice versa can be derived, but not for the Extended RNA code type 1, as its complementary is a hyperprism in which the Hamming distance between some codons is not 1, but rather 3 (José et al. 2007). When the symmetry groups have a structure-preserving one-to-one correspondence are considered isomorphic (Sethuraman 1997; Artin 1991; Weisstein 2022).

#### Remark

Regarding the symmetries, the RNY code and the whole SGC display a primitive algebraic structure known as the Four-Klein Group, which is the only non-cyclic group. This group can be envisaged as the minimum algebraic structure to build a genetic code with 4 nucleotides. The Four-Klein Group is an Abelian (commutative) group in which every element, different of the neutral, has order 2. Every element is its own inverse, and the sums of complementary RNA/DNA bases, the so-called Watson–Crick base pairs, are constant. All the Klein four-groups are pairwise isomorphic. The Four-Klein Group is the smallest finite group that is not cyclic, but it arises from the direct product of 2 binary cyclic groups. Indeed, the direct product  where  is a binary group, with addition operation modulo 2, is its canonical group theoretical representation. Every binary set that contains the null element defines a binary subgroup. It means that the two classes of any of the three binary partitions (purines-pyrimidines, strong–weak, and amino-keto) are the member classes of the factor group over the binary subgroup that contains the null element. Further mathematical properties of the Four-Klein Group useful for the representation of the genetic code can be found elsewhere (José et al. 2012).

## Results

**Fig. 1:**
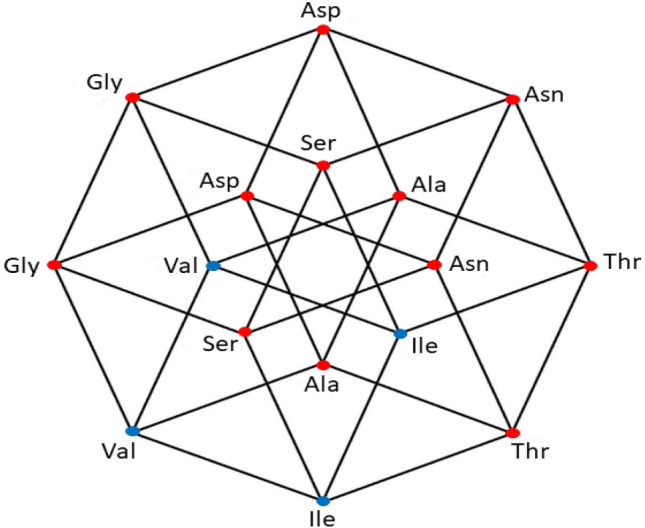
aaRSs distribution in code RNY. Class I (blue, 2 aminoacids) and Class II (red, 6 aminoacids). Graphic representation of the subsets RYY and RRY. First 4-dimensional hypercube of the RNY code: RNY =RYY U RYY

**Fig. 2:**
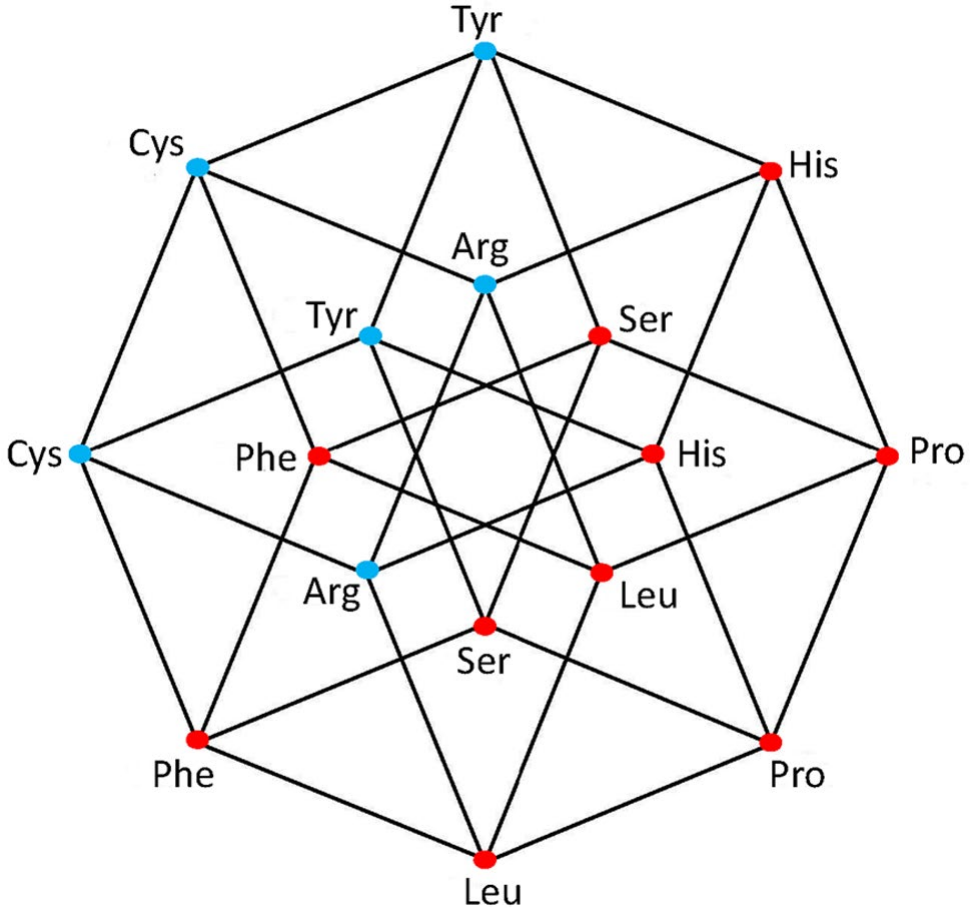
aaRSs distribution in the YNY code. Class I (blue, 3 aminoacids) and Class II (red, 5 aminoacids). Graphic representation of the subsets YYY and YRY. Second 4-dimensional hypercube of the Extended RNA-code type 2: ${\text{YNY}} = {\text{YYY}} \cup {\text{YRY}}$

**Fig. 3:**
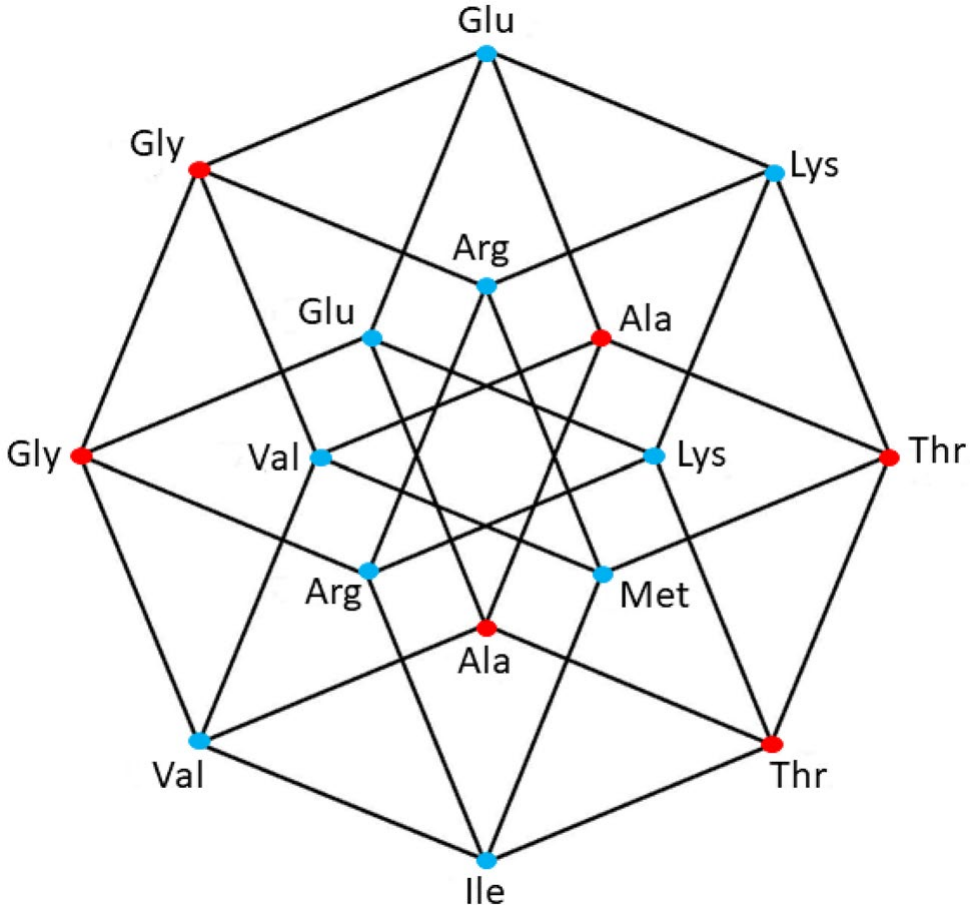
aaRSs distribution in the RNR code. Class I (blue, 5 aminoacids) and Class II (red, 3 aminoacids). Graphic representation of the subsets RYR and RRR. Third 4-dimensional hypercube of the Extended RNA-code type 2: ${\text{RNR}} = {\text{RYR}} \cup {\text{RRR}}$

**Fig. 4:**
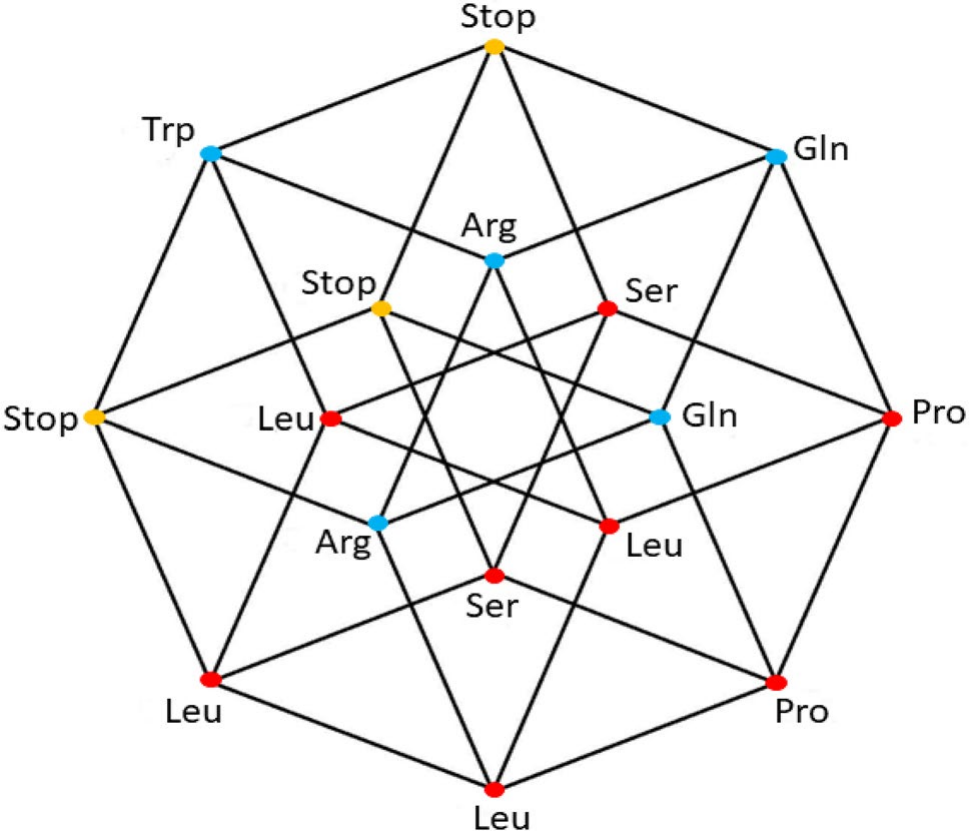
aaRSs distribution in the YNR code. Class I (blue, 3 aminoacids) and Class II (red, 3 aminoacids). Graphical representation of the subsets YYR and YRR. Note the three stop codons (yellow vertexes). Fourth 4-dimensional hypercube of the Extended RNA-code type 2:${\text{YNR}} = {\text{YYR}} \cup {\text{YRR}}$

**Fig. 5:**
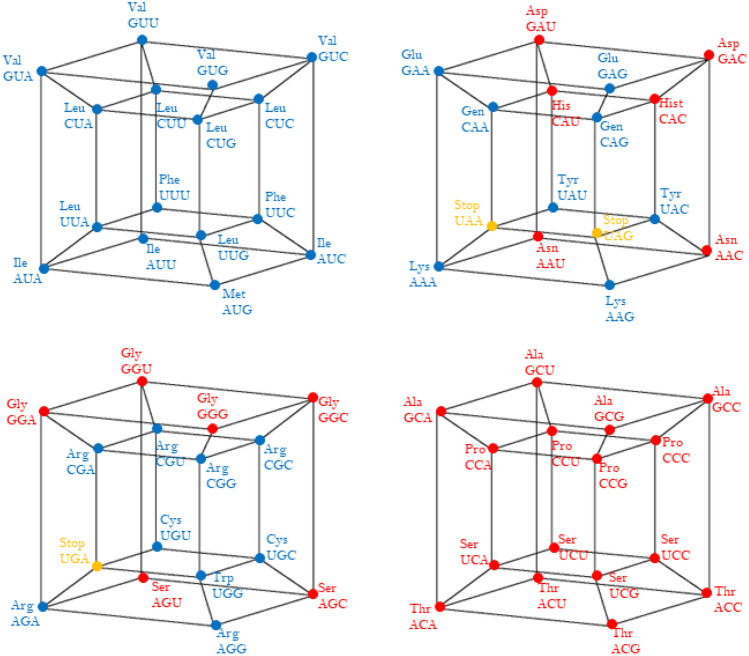
aaRSs distribution in Standard Genetic Code of Class I (blue) and Class II (red)

All hypercubes shown in Figs. 1, 2, 3, 4, 5 are calculated using MATLAB R12.

### The extended RNA code type II and its three main sets of triplets

The distribution of both Classes of aaRSs assuming a RNY code is shown in the 4-dimensional hypercube in Fig. 1. Note that ValRS and IleRs belong to Class Ia, and GlyRS, SerRS, ThrRS, AlaRS pertain to Class IIa, and AspRS, AsnRs are members of Class IIb. The set of codons of the form RNY constitutes a 4-dimensional hypercube, which is a coordinated affine subspace (i.e., a translation), or a 4-dimensional hyperface of the 6D hypercube of the SGC.

A primaeval RNA world was proposed which comprises the codons with an RNY pattern. These 16 codons code for eight amino acids, having every amino acid two triplets that specify it. Allowing transversions in the first or in the third nucleotide base the YNR and RNR patterns emerge. *These 3 patterns altogether are here defined as the codons of the Extended RNA code type 2*. This extended code includes 48 triplets which specify 18 amino acids, including AUG, the start codon, but not the 3 stop codons. The remaining 16 codons, the YNR triplets, can only be obtained by double transversions in the RNY triplets, occurring simultaneously, in the first and the third nucleotide base. The first set corresponds to the so-called primaeval RNA code (RNY pattern); the second (YNY pattern) and the third (RNR pattern) sets correspond to triplets obtained by transversion in the first nucleotide or in the third nucleotide, respectively. The fourth set (YNR pattern) corresponds to triplets obtained by transversions, occurring simultaneously in the first and in the third nucleotide. Each of these 4 sets can be partitioned into 2 subsets by replacing the N by Y or R. Hence, the set of RNY triplets is the union of the set of RYY triplets with the set of RRY. Analogously, the set of YNY triplets is the union of the YYY with the YRY triplets, while the set of RNR triplets is the union of the RYR with the RRR triplets. Finally, the set of YNR triplets is the union of the YYR with the YRR triplets. These 8 sets conform a partition of the set of 64 triplets, having each of them 8 elements. In fact, each one of these 8 sets is a 3-dimensional coordinated affine subspace (Coxeter, 19,873), that is, a cube, contained in the 6-dimensional hypercube. This is so since the *Hamming distance* (e.g., José et al. 2010) between triplets on the same edge is 1.

### The second set of the extended RNA code type 2

The 16 YNY triplets can be obtained from the triplets of the primaeval RNA code, by transversion in the first nucleotide (Fig. 2). Note that a 4D-hypercube is again obtained, i.e., this hypercube is invariant under the transversions. This invariance means that the transformation is symmetric. The set of triplets of the form YNY is a disjoint set with the set of the RNY. The 16 triplets of the form YNY specify 8 amino acids, one of which (Ser) was already found amongst those coded by the RNY triplets. Then, the triplets of the form YNY give rise to 7 new amino acids. The set of codons of the form YNY is the union of the cubes YYY and YRY in which every amino acid corresponds to an edge of the associated cube. We consider the union of the sets of codons of the form RNY and YNY, that is the set of the NNY triplets and their correspondence with their 15 amino acids, as an extension of the original RNY code. The set of the 32 NNY triplets, that is, those which end in pyrimidines, is a 5-dimensional vector subspace of the 6-dimensional hypercube.

### The third set of the extended RNA code type II

The 16 RNR triplets can be obtained from the triplets of the primaeval RNA code, by transversion in the third nucleotide. Again, a 4D-hypercube is obtained, which means a symmetric transformation. The set of triplets of the form RNR is a disjoint set with the set of the RNY. The 16 triplets of the form RNR specify 9 amino acids, 6 of which were already found amongst those coded by the NNY triplets. Then, the triplets of the form RNR give rise to only 3 new amino acids. The set of codons of the form RNR is the union of the cubes RYR and RRR. Note that in the cube RYR (Fig. 3), 3 amino acids correspond to 3 out of the 12 solid edges of the cube, but there are 2 amino acids, (Met and Ile), that are associated, each of them, to only one vertex. The set of codons of the form RNR conforms a 4-dimensional hypercube, which is a coordinated affine subspace (Coxeter 1973), or a 4-dimensional hyperface of the 6-dimensional hypercube of the 64 triplets.

### The fourth set of the extended RNA code type II

The 16 YNR triplets can be obtained from the triplets of the primaeval RNA code, by simultaneous transversions in the first and the third nucleotides. The set of triplets of the form YNR is also a disjoint set with the set of the RNY. The 16 triplets of the form YNR specify 6 amino acids, 4 of which were already found amongst those coded by the NNY triplets. Then, the triplets of the form YNR give rise to only 2 new amino acids. In Fig. 4, there are also 2 amino acids that correspond to 2 solid edges and 1 amino acid (Trp) associated with only one vertex. In Fig. 4**,** there are also 3 stop codons, located in 2 connected edges of the cube. It means that the set of codons of the form RNR conforms a 4-dimensional hypercube, which is a coordinated affine subspace, or a 4-dimensional hyperface of the 6-dimensional hypercube of the 64 triplets. Now we observe that the union of the sets RNR and YNR, that is the set of the NNR triplets, those that end in purines, is a 5-dimensional affine subspace, or a 5-dimensional hypercube, whose associated vector subspace is that of the NNY triplets. The hypercube of the NNR triplets specify only 5 new amino acids, not specified by the NNY triplets. They are the amino acids whose canonical triplets end in purines, A or G.

A note of caution: all the hypercubes presented in Figs. 1, 2, 3, 4, 5 are orthotopes, where all the angles between adjacent edges are right angles, but they may appear like acute or obtuse.

The distribution of both Classes of aaRSs assuming the Extended RNA code type 1 and type 2 will each form a 5-dimensional hypercube (not shown). New synthetases appear in this Extended RNA code type 1, to wit, LeuRS, CystRS, ArgRS, and MetRS that belong to Class1a, GlnRS is of Class 1b, TrpRS and TyrRS are members of Class 1c, and HisRS pertains to Class 2a. In the Extended RNA code type 2, new aaRSs appear: GluRS is of Class 1b, ProRS is of Class 2a, PheRS pertains to Class 2c, and LysRS can be Class 1b or Class 2b. Stop codons (UAA, UAG, UGA) appear only in the Extended RNA code type 1 and are included for determining the symmetry groups. Stop codons are also used in mitochondria and chloroplasts.

The distribution of both Classes of aaRSs assuming the SGC is shown in Fig. 5. The SGC is a 6D hypercube formed by four 4D hypercubes. Remember that the aaRSs of Class I are mirror images of the aaRSs of Class II, a characteristic only seen in the SGC.

## Discussion

In this work, we have determined and analyzed the distribution of aaRSs for different genetic codes: RNY, Extended RNA code type 1 and type 2, and the SGC. We represented each code by their corresponding polytopes (hypercubes), and we have outlined the type of symmetries for both Classes of aaRSs. All synthetases of Class II can be found in the first two 4D-hypercubes (RNY and YNY). Also note that Class I and II of aaRSs existed for all 20 amino acids in the Extended RNA codes 1 and 2, before the completion of the SGC. The 20 amino acids plus the stop codons were already present when both Extended codes are considered. This agrees with the proteomes before LUCA (Farías et al. 2016; Palacios-Pérez and José, 2019) which are like the proteome of LUCA (Weiss et al. 2016). According to the symmetries found in the last step to arrive to the SGC, duplications of Lys, Arg, Glu, Gly, Pro, Leu, Ser, and Phe for the Extended RNA code type 1, and duplications of Gln, Arg, Stop, Trp, Pro, Leu, and Ser for the Extended RNA code type 2, were necessary, resulting in a simpler algebraic structure of the SGC (see Table 1).

The hypercubes consider mono-codonic, di-codonic, three-codonic, tetra-codonic, and hexa-codonic amino acids. In each code, we can observe symmetrical structures of the distribution of aaRSs. We observed that the symmetrical properties of the aaRSs distribution in the SGC are simpler than the ones observed for both Extended RNA codes.

Also notice the gradual presence of SerRS (Class II), which does not recognize the anticodon of tRNA^Ser^ but instead recognizes the variable arm found in all tRNA^Ser^ isoacceptors (Biou et al. 1994; Vincent et al. 1995). This would be expected for an enzyme whose substrate is a set of tRNAs that must recognize six different codons (Leu, Ser and Arg), four (Gly, Pro, Thr, Val, Ala), three (Ile), and two (Cys, Glu, Lys, Gln, His, Phe, Asn, Asp). There is only one aaRS for each mono-codonic amino acids (Met, Trp, and Tyr). In short, there are only 20 aaRSs, one for each amino acid and, respectively, for isoacceptor tRNAs; hence, the aaRS link to the 20 coded amino acids is non-degenerate (Eriani et al. 1900).

Note from the distribution of aaRSs in both Extended RNA code type I and II, and the SGC, are symmetrical, which is consistent with the notion that the evolution of the two aaRS classes evolved in parallel (Eriani et al. 1990; Schimmel et al. 1993; Ribas de Pouplana and Schimmel 2001a, 2001b).

The split of the RO model divides all aaRSs into two symmetrical (10 + 10) groups, which correspond to the I and II classes of aaRSs, as can be seen in the hypercube in Fig. 5. This equality is made possible by double-strand coding (Rodin and Rodin 2006; Rodin et al. 2009). In our analysis, there is an enormous gap between the root of aaRS, presumably a sense-antisense gene, and the RNY code. It has been proposed that in the early stages of the history of life, protein or peptide synthesis was established by RNA, i.e., ribozymes (Wolf and Koonin 2007). Indeed, it has been demonstrated that a ribozyme can attach an activated amino acid to a tRNA (Wolf and Koonin 2007; Piccirilli et al. 1992; Illangasekare et al. 1995; Carter and Wolfenden 2015; Li et al. 2013). These results imply that in the early stages of the evolution of the translation system, protein or peptide synthesis could be performed by a translation system using a ribozyme without the need of proteinaceous aaRSs.

The main difference between the amino acids recognized by the two aaRS Classes is their average size (Carter et al. 2019). Class I and II aminoacyl-tRNA synthetase tRNA groove discrimination created the first synthetase•tRNA cognate pairs and was therefore essential for the formation of genetic coding (Carter et al. 2019). Our group-theoretical approach does not explicitly consider how the allocation of aaRSs during the evolution of the genetic code was constrained by structural and functional properties of the interaction of aaRS, amino acids, and tRNA. Yet it permits us to determine the types of symmetries of aaRSs during the evolution of the SGC. The symmetry groups found in the RNA codes pinpoint to intricacies in the evolution of aaRSs in conjunction with the evolution of the genetic code itself. The biological usefulness of the results emphasizes the roles of extensive occurrence of aaRS gene duplication involving every synthetase family, Horizontal Gene Transfer (including concessions, swapping of genes, recombination events), and a Lamarckian mode of inheritance for the diversification and innovation of synthetases (Vetsigian et al. 2006; Krahn et al. 2022; Rubio et al. 2015).
